# Nicotine-induced activation of cholinergic receptor nicotinic alpha 5 subunit mediates the malignant behaviours of laryngeal squamous epithelial cells by interacting with RABL6

**DOI:** 10.1038/s41420-024-02051-x

**Published:** 2024-06-15

**Authors:** Yujie Shen, Qiang Huang, Xiaohui Yuan, Hongli Gong, Chengzhi Xu, Huaidong Du, Chi-Yao Hsueh, Liang Zhou

**Affiliations:** Department of Otorhinolaryngology, Eye & ENT Hospital, Fudan University, Shanghai, 200031 China

**Keywords:** Prognostic markers, Head and neck cancer

## Abstract

Nicotine, a crucial constituent of tobacco smoke, can bind to and activate nicotinic acetylcholine receptors (nAChRs), thereby regulating various biological functions. However, the specific mechanisms through which nicotine mediates nAChRs to regulate the metastasis of laryngeal squamous cell carcinoma (LSCC) remain elusive. In this study, smoking status was found to be closely associated with metastasis in patients with LSCC. In addition, nicotine exposure potentiated the hematogenous and lymphatic metastatic capacity of LSCC cells. Nicotine activates membrane-bound CHRNA5, promoting cell migration and invasion, EMT and cell–ECM adhesion in LSCC. Furthermore, this study demonstrated that the Ras superfamily protein RABL6 directly interacted with CHRNA5, which preferentially binds to the RABL6-39-279aa region, and this interaction was enhanced by nicotine. Nicotine-mediated activation of CHRNA5 enhanced its interaction with RABL6, triggering the JAK2/STAT3 signalling pathway and eventually augmenting the metastatic potential of LSCC cells. This study reveals a novel mechanism through which nicotine-mediated CHRNA5–RABL6 interaction promotes the metastasis of LSCC. The findings of this study may help to develop effective strategies for improving the outcome of patients with LSCC in clinical settings.

## Introduction

Head and neck squamous cell carcinoma (HNSCC) encompasses malignant tumours that originate from the larynx, oral cavity, nasal cavity, pharynx and other associated anatomical structures [1]. Epidemiological data suggest that HNSCC is the sixth most common cancer worldwide, and its incidence is progressively increasing [2]. Laryngeal squamous cell carcinoma (LSCC) accounts for over 95% of all malignant laryngeal cancers, making it one of the most common types of squamous cell carcinoma in the head and neck region [3]. Early symptoms of LSCC are not specific and resemble those of other laryngeal diseases, often leading to challenges in timely diagnosis. Consequently, the majority of patients are diagnosed in the intermediate to advanced stages of LSCC, which frequently results in death from recurrence and/or metastasis, and is associated with a poor prognosis [4]. Therefore, identifying potent biomarkers is of paramount clinical significance for early detection and individualised treatment of LSCC and for improving patient outcomes.

The pathogenesis of LSCC is multifaceted and involves various factors, including smoking, alcohol consumption, malnutrition, dysregulation of the immune system and exposure to noxious substances [1]. Notably, smoking remains the primary risk factor for LSCC. Epidemiological studies have demonstrated that the risk of LSCC in smokers is approximately 10-fold higher than that in non-smokers, with 70–80% of LSCC cases being attributed to smoking and alcohol consumption [5].

Nicotine, a crucial constituent of cigarette smoke and emerging electronic cigarettes, has attracted extensive attention from researchers. It is a natural alkaloid primarily present in the Solanaceae plant family [6] and is classified as an N-alkylpyridine owing to its nitrogen-containing aromatic ring and hexacyclic structure [7]. N-alkylpyridine compounds interact with acetylcholine receptors in the body, resulting in various biological effects [8]. To date, 16 subtypes of nicotinic acetylcholine receptors (nAChRs) have been identified in human tissues, including α1–α7, α9, α10, β1–β4, γ, δ and ε subunits [9]. However, the molecular mechanisms through which nicotine triggers nAChRs and modulates the pathogenesis, progression, and metastasis of LSCC remain unclear.

In this study, nicotine-induced upregulation of CHRNA5 was found to promote the metastatic potential of LSCC cells in vitro and in vivo, and CHRNA5 was found to be significantly associated with lymph node metastasis and poor prognosis in patients with LSCC. In addition, CHRNA5 directly interacted with the RABL6 protein, and activation of CHRNA5 by nicotine upregulated RABL6 expression and subsequently triggered the JAK2/STAT3 signalling pathway to enhance the migratory and invasive capabilities of LSCC cells.

## Results

### Smoking was associated with a higher incidence of lymph node metastasis and poor prognosis in patients with HNSCC

**Fig. 1:**
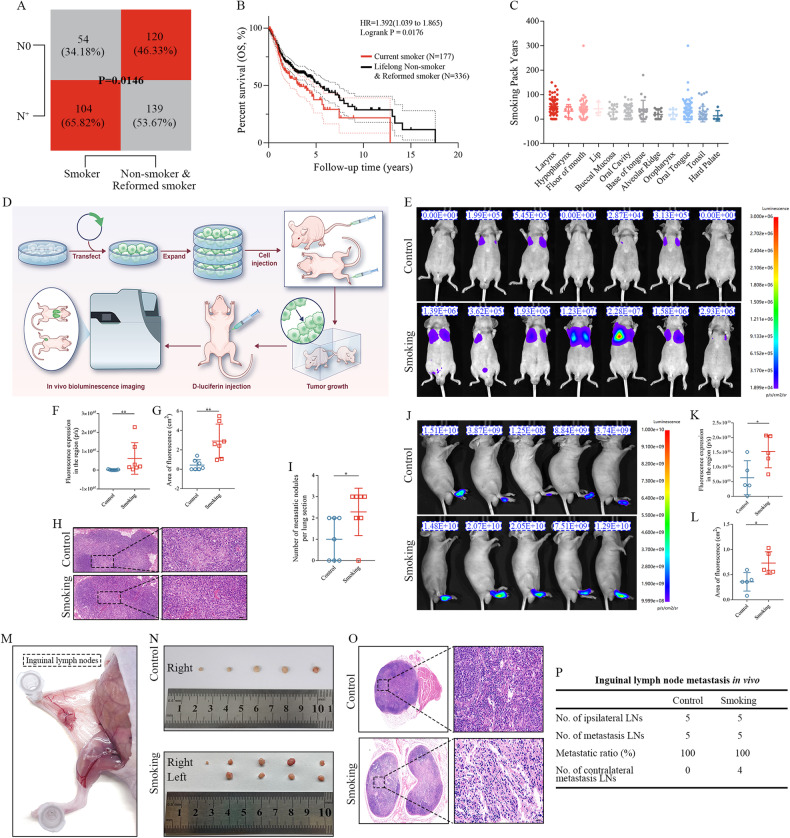
Cigarette smoke enhanced the haematogenous and lymphatic metastatic capabilities of LSCC cells. **A** Patients with HNSCC with a smoking history had a higher incidence of lymph node metastasis. **B** The prognosis of current smokers was poorer than that of lifelong non-smokers and reformed smokers. **C** The number of pack-years was higher among patients with laryngeal cancer than among those with other types of HNSCC. **D** Schematic representation of the establishment of mouse models of tail vein metastasis and inguinal lymph node metastasis. **E** Bioluminescence images of the lungs of nude mice injected with luciferase-labelled AMC-HN8 cells into the tail vein. The fluorescence intensity (**F**) and fluorescence area **G** of lung tissue sections were higher in the smoking group than in the control group. **H** Representative images of HE staining of lung tissue sections in the control and smoking groups. Scale bar: 20 μm. **I** The number of pulmonary metastatic nodules was higher in the smoking group than in the control group. **J** Bioluminescence images of the right lower limbs of nude mice injected with luciferase-labelled AMC-HN8 cells into the footpad. The fluorescence intensity (**K**) and fluorescence area (**L**) of the right lower limb were higher in the smoking group than in the control group. **M** Representative images of the inguinal lymph node metastasis model. **N** Representative images of enucleated inguinal lymph nodes. **O** Representative images of HE staining of inguinal lymph nodes. Scale bar: 20 μm. **P** Metastatic ratio of inguinal lymph nodes.

Data extracted from TCGA cohort were retrospectively analysed. The results of chi-squared test revealed that the incidence of lymph node metastasis was higher among current smokers than among lifelong non-smokers and reformed smokers (*p* = 0.0146; Fig. 1A). KM analysis revealed that the 5-year overall survival (OS) rate of patients with HNSCC was approximately 50% (Fig. 1B). In particular, the OS of current smokers was markedly worse than that of lifelong non-smokers and reformed smokers (*p* = 0.0176; Fig. 1B). Furthermore, the number of pack-years (calculated as smoking years multiplied by packs/day) was significantly higher among patients with laryngeal cancer than among patients with other types of HNSCC (Fig. 1C), indicating that smoking has a specific impact on the prognosis of LSCC.

### Cigarette smoke enhanced the haematogenous and lymphatic metastasis of LSCC

Luciferase-labelled AMC-HN8 cells were produced through lentiviral infection (Supplementary Fig. 1A). To examine the effects of smoking on the metastasis of LSCC, we established mouse models of tail vein metastasis and inguinal lymph node metastasis using luciferase-labelled AMC-HN8 cells (*n* = 7 mice/group, Fig. 1D). BLI was performed on nude mice after 4 weeks of tail vein injection to monitor the metastatic potential of LSCC cells (Fig. 1E). The fluorescence intensity and fluorescence area were substantially higher in the smoking group than in the control group (*p* < 0.01; Fig. 1F, G). After 8 weeks, the mice were euthanised and their lungs were excised. Histological analysis of lung sections stained with H&E (Fig. 1H) revealed a significantly higher number of pulmonary metastatic nodules in the smoking group (*p* < 0.05; Fig. 1I).

Furthermore, mouse models of inguinal lymph node metastasis were used to investigate the impact of smoking on lymph node metastasis of LSCC. Luciferase-labelled AMC-HN8 cells were injected into the foot pads of mice (*n* = 5 mice/group). Smoking enhanced the lymph node metastasis of LSCC as evidenced by the fluorescence intensity and area of metastatic lymph nodes (*p* < 0.05; Fig. 1J–L). As depicted in Fig. 1M, the mice were sacrificed and both lower limbs were dissected after 8 weeks of injection. Enlarged and suspicious lymph nodes were observed in the contralateral inguinal region in the smoking group (Fig. 1N–P). These lymph nodes were identified to be metastatic via H&E staining (Fig. 1O). In addition, the number of pan-cytokeratin-positive tumour cells in inguinal lymph nodes was higher in the smoking group than in the control group (Supplementary Fig. 1B). Altogether, these findings suggest that smoking contributes to the hematogenous and lymphatic metastatic capacity of LSCC cells.

### Nicotine facilitated the malignant metastasis of LSCC and upregulates CHRNA5

**Fig. 2:**
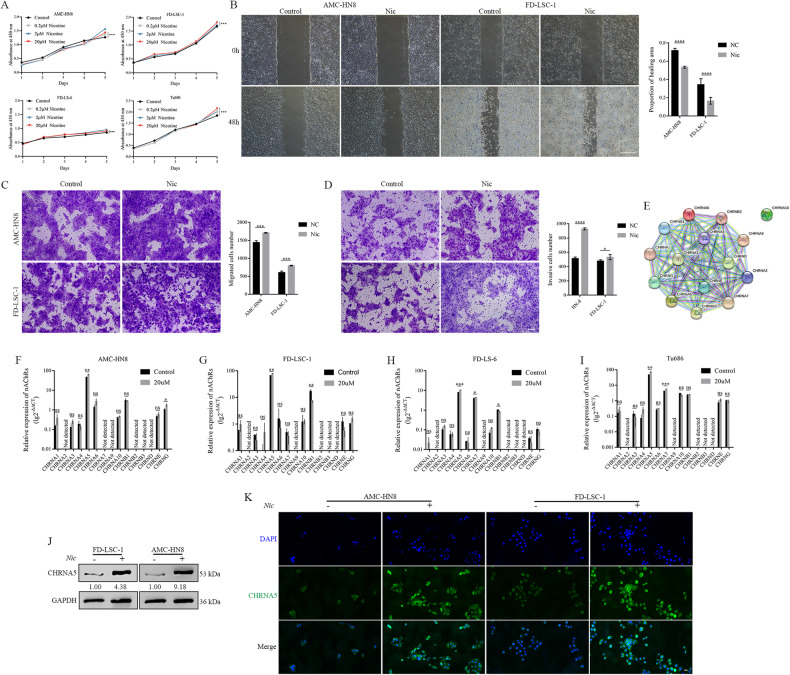
Nicotine facilitated the malignant metastasis of LSCC by upregulating CHRNA5. **A** CCK-8 assay was performed in four LSCC cell lines cultured with varying concentrations of nicotine (0.2–20 μM). **B** Wound healing assay revealed that 20-μM nicotine enhanced the proliferation of LSCC cells. Scale bar: 500 μm. Transwell migration (**C**) and invasion (**D**) assays demonstrated that 20-μM nicotine facilitated the metastasis of LSCC cells. Scale bar: 100 μm. **E** Protein–protein interaction network of nAChRs. Expression of nAChRs following nicotine stimulation in AMC-HN8 (**F**), FD-LSC-1 (**G**), FD-LS-6 (**H**) and Tu686 (**I**) cells. (**J**) Protein expression of CHRNA5 after nicotine stimulation. GAPDH served as a loading control. **K** Immunofluorescence staining revealed that CHRNA5 (green) was primarily localised in the cell membrane and cytoplasm and that nicotine upregulated CHRNA5 (green) in LSCC cells. Scale bar: 20 μm.

As the crucial constituent of cigarette smoke and e-cigarettes, nicotine has been identified as a significant risk factor for the development and progression of cancer [6]. To determine the optimal dose of nicotine stimulation, the viability of LSCC cells was evaluated after they were treated with varying concentrations of nicotine (0.2–20 μM) for 5 days. Nicotine at the concentration of 20 μM significantly enhanced the proliferative, migratory and invasive capabilities of LSCC cells (Fig. 2A–D), which is consistent with the results of previous studies [10–14]. In addition, in LSCC cells, nicotine decreased the protein expression of the EMT markers E-cad and ZO-1 and increased the protein expression of N-cad, another EMT marker (Supplementary Fig. 1C). Altogether, we established an in vitro model of nicotine-induced metastasis and demonstrated that nicotine promotes the metastatic potential of LSCC cells.

Tobacco smoking results in nicotine exposure, which exerts its effects by binding to nAChRs [15] (Fig. 2E). Multiple nAChRs were expressed in LSCC cells; however, CHRNA2, CHRNA9, CHRNB2, CHRNB3 and CHRND were not detected (Fig. 2F–I). After nicotine stimulation, CHRNA7, CHRNB1 and CHRNG were upregulated in some LSCC cell lines, whereas CHRNA5 was significantly upregulated in all tested LSCC cell lines (Fig. 2F–I), which was consistent with the high protein expression of CHRNA5 (Fig. 2J). Nicotine-induced upregulation of CHRNA5 was mainly observed in the cell membrane and cytoplasm (Supplementary Fig. 1D). Immunofluorescence analysis validated that CHRNA5 was primarily localised in the cell membrane and cytoplasm and that nicotine upregulated CHRNA5 expression in LSCC cells (Fig. 2K).

### Nicotine promoted the metastasis of LSCC by activating CHRNA5

**Fig. 3:**
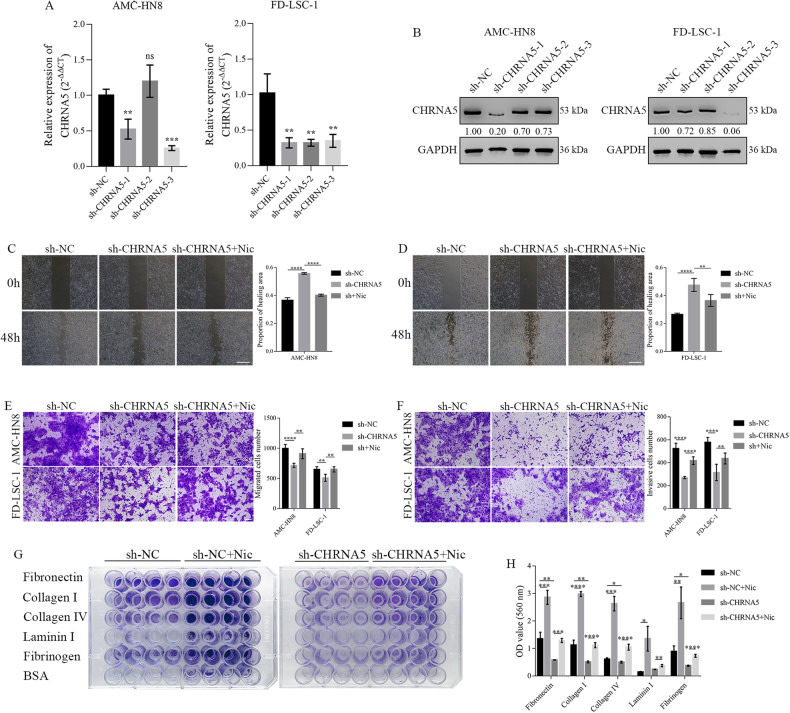
Nicotine promoted the metastasis of LSCC by activating CHRNA5 in vitro. qRT-PCR (**A**) and western blotting (**B**) were performed to examine the mRNA and protein expression of CHRNA5, respectively, in AMC-HN8 and FD-LSC-1 cells infected with sh‐control, sh‐CHRNA5-1, sh‐CHRNA5-2 and sh‐CHRNA5-3 lentiviruses. Wound healing assay (**C**, **D**, Scale bar: 500 μm) and transwell assay (**E**, **F**, Scale bar: 100 μm) demonstrated that CHRNA5 knockdown significantly inhibited the migratory and invasive capabilities of LSCC cells; however, these effects were reversed by nicotine. Representative images (**G**) and quantitative analysis (**H**) of cell adhesion assay, which demonstrated that CHRNA5 knockdown effectively inhibited the adhesion of LSCC cells to the extracellular matrix; however, these results were reversed by nicotine.

To investigate the function of CHRNA5, sh-NC and sh-CHRNA5 lentiviruses were infected into AMC-HN8 and FD-LSC-1 cells. qRT-PCR and western blotting were performed to verify the knockdown efficiency (Fig. 3A, B). Based on the results, AMC-HN8 cells infected with sh-CHRNA5-1 lentivirus and FD-LSC-1 cells infected with sh-CHRNA5-3 lentivirus were selected for further experiments. GFP fluorescence was analysed to assess infection efficiency (Supplementary Fig. 1E). Wound healing assay (Fig. 3C, D) revealed that CHRNA5 knockdown significantly suppressed the healing capability of AMC-HN8 and FD-LSC-1 cells, whereas nicotine treatment enhanced the healing capability to some extent. Transwell assay revealed that CHRNA5 knockdown inhibited the migratory (Fig. 3E) and invasive (Fig. 3F) capabilities of LSCC cells; however, nicotine treatment reversed these effects. CHRNA5 knockdown increased E-cadherin and ZO-1 expression but decreased N-cadherin expression in LSCC cells; however, nicotine treatment reversed these effects (Supplementary Fig. 1F). These results suggest that CHRNA5 enhances the migratory and invasive capabilities of nicotine-stimulated LSCC cells by promoting EMT. Metastasis is a complex process in which cancer cells detach from the primary tumour, infiltrate surrounding tissues and anchor themselves at a distant site. During tumour metastasis, tumour cells enter the bloodstream or lymphatic system from the primary tumour and subsequently invade the tissues of the target organ. During this process, strengthening the adhesion ability can increase the adhesion of tumour cells to the vascular endothelium, making it easier for them to invade the target tissue and increase the likelihood of successful colonisation. Cell adhesive ability was determined using the CytoSelect™ 48-well Cell Adhesion Assay. The results revealed that nicotine significantly enhanced the adhesion of LSCC cells to the ECM proteins Fibronectin, Collagen I, Collagen IV, Laminin I and Fibrinogen (Fig. 3G, H). CHRNA5 knockdown effectively inhibited the adhesion of LSCC cells to the ECM (compared with the sh-NC [control] group); however, these changes were reversed by nicotine (Fig. 3G, H). These findings suggest that ECM proteins may bind to the cell surface receptor CHRNA5, which is activated by nicotine, and hence facilitate the distant anchoring of LSCC cells during metastasis.

**Fig. 4:**
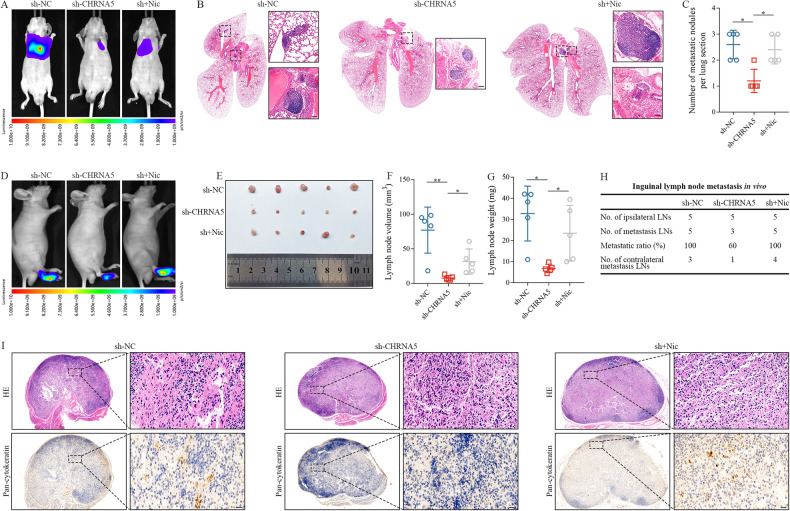
Nicotine promoted the metastasis of LSCC by activating CHRNA5 in vivo. Luciferase-labelled AMC-HN8 cells stably expressing sh-CHRNA5 and sh-NC were injected into the tail veins and footpad of mice to construct tail vein metastasis and inguinal lymph node metastasis models, respectively. **A** Representative bioluminescence images of the lungs of nude mice treated with sh-NC, sh-CHRNA5 and sh-CHRNA5 after nicotine stimulation. **B** Representative HE staining of the lung tissue sections of mice. Scale bar: 100 μm. **C** Histogram analysis of the number of pulmonary metastatic nodules. **D** Representative bioluminescence images of the right lower limbs of nude mice treated with sh-NC, sh-CHRNA5 and sh-CHRNA5 after nicotine stimulation. **E** Representative images of the gross appearance of inguinal lymph nodes in nude mice. Measured lymph node volumes (**F**) and weights (**G**). **H** Metastatic ratio of inguinal lymph nodes. **I** Representative images of HE and IHC staining of inguinal lymph nodes with pan-cytokeratin. Scale bar: 20 μm.

To examine the effects of CHRNA5 on nicotine-induced LSCC metastasis in vivo, we established mouse models of lung colonisation (*n* = 5 mice/group). Nude mice were imaged serially via BLI at week 4 after injection (Fig. 4A) and sacrificed at week 8 after injection (Supplementary Fig. 1G). HE staining of lung tissue sections showed that mice in the sh-CHRNA5 group had fewer pulmonary metastatic nodules than those in the sh-NC and sh-CHRNA5 + nicotine groups (*p* < 0.05; Fig. 4B, C). Furthermore, we investigated the role of CHRNA5 in lymphatic metastasis (*n* = 5 mice/group; Fig. 4D). Mice in the sh-CHRNA5 group had smaller and lighter lymph nodes than those in the sh-NC and sh-CHRNA5 + nicotine groups (*p* < 0.05; Fig. 4E–G). In addition, mice in the sh-CHRNA5 group had a lower inguinal lymph node metastasis ratio and fewer contralateral metastatic lymph nodes than mice in the sh-NC and sh-CHRNA5 + nicotine groups (Fig. 4H). Moreover, mice in the sh-CHRNA5 group had fewer pan-cytokeratin-positive tumour cells in the inguinal lymph nodes than mice in the sh-NC and sh-CHRNA5 + nicotine groups (Fig. 4I). Altogether, these findings indicate that nicotine promotes LSCC metastasis by activating CHRNA5.

### High CHRNA5 expression was associated with a poor prognosis in patients with LSCC who smoked

**Fig. 5:**
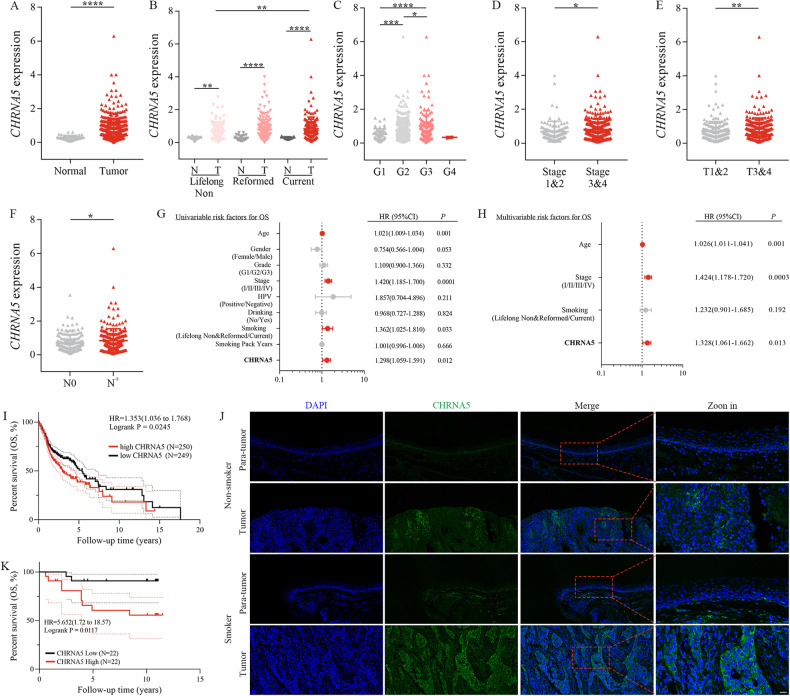
CHRNA5 overexpression was associated with the metastasis and poor prognosis of LSCC. **A** Differential expression analysis of CHRNA5 in TCGA cohort. Differential expression analysis of CHRNA5 in patients with LSCC stratified based on the smoking status (**B**), tumour grade (**C**), TNM stage (**D**), T stage (**E**) and N stage (**F**) in TCGA cohort. Univariate (**G**) and multivariate (**H**) Cox regression analyses were performed to examine the OS of patients in TCGA cohort. **I** Kaplan–Meier curves were plotted to compare the OS of patients with low and high CHRNA5 expression in TCGA cohort. The median expression was used as the cut-off value. **J** Representative images of immunofluorescence staining demonstrating that CHRNA5 (green) was higher in LSCC tissues (smoking group) than in adjacent normal tissues in cohort 2. Scale bar: 20 μm. **K** Kaplan–Meier curves were plotted to compare the OS of patients with low and high CHRNA5 expression in cohort 2. The median expression was used as the cut-off value.

In TCGA cohort, CHRNA5 expression was higher in neoplastic tissues than in adjacent normal tissues (*p* < 0.0001; Fig. 5A). The same results were observed in three cohorts comprising patients with different smoking statuses (Fig. 5B). CHRNA5 expression was higher in neoplastic tissues of current smokers than that of lifelong non-smokers (*p* < 0.01; Fig. 5B) and increased with increasing grade (*p* < 0.05; Fig. 5C), TNM stage (*p* < 0.05; Fig. 5D) and tumour size (*p* < 0.01; Fig. 5E). In addition, CHRNA5 expression was higher among patients with lymph node metastasis (N^+^) than among those without lymph node invasion (N0) (*p* < 0.05; Fig. 5F). Based on the results of univariate and multivariate Cox regression analyses, CHRNA5, age and TNM stage were identified as independent prognostic factors in TCGA cohort (Fig. 5G, H). Survival analysis revealed that high CHRNA5 expression was associated with a lower overall survival rate in patients in TCGA cohort (*p* = 0.0245; Fig. 5I). To verify these findings, we collected and tested 50 fresh LSCC tissues and paired adjacent normal tissues (cohort 1, *n* = 50). Consistently, CHRNA5 expression was higher in LSCC tissues than in paired adjacent normal tissues (*p* < 0.05; Supplementary Fig. 2A), and this phenomenon was mainly observed in the smoking group (*p* < 0.001; Supplementary Fig. 2B). In addition, CHRNA5 expression in LSCC tissues was positively correlated with the number of pack-years (*p* = 0.0455; Supplementary Fig. 2C). Furthermore, 44 paraffin-embedded LSCC tissue sections (cohort 2, *n* = 44) were collected and tested via immunofluorescence staining. As shown in Fig. 5J, the CHRNA5 protein (in green) was mainly localised in the cell membrane and was upregulated in LSCC tissues compared with adjacent normal tissues. In particular, CHRNA5 protein expression was higher in smokers than in non-smokers (Fig. 5J). Fluorescence intensity was calculated using the Image J software, and patients in cohort 2 were divided into high- and low-expression groups based on the median fluorescence value of the continuous variable. High expression of CHRNA5 was associated with a lower overall survival rate in patients with LSCC (*p* = 0.0117; Fig. 5K), which was consistent with the results of survival analysis in TCGA cohort. Altogether, these results suggest that CHRNA5 is highly expressed in patients with LSCC, especially those who smoke, and indicates a worse prognosis.

### Nicotine-induced activation of CHRNA5 promoted RABL6 expression to facilitate LSCC metastasis

**Fig. 6:**
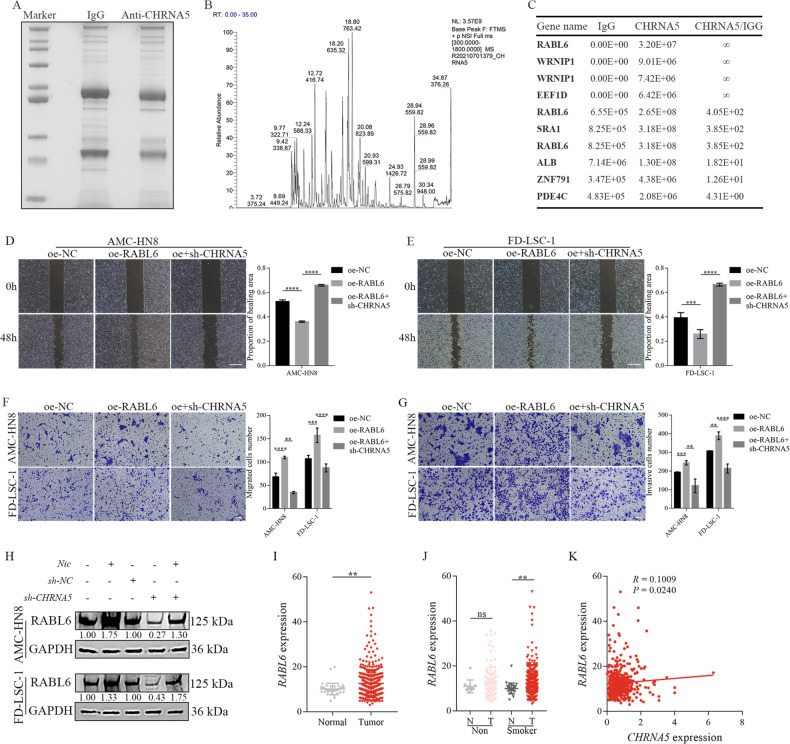
Nicotine-induced activation of CHRNA5 promoted RABL6 expression to facilitate the metastasis of LSCC. **A** The immunoprecipitated proteins were separated via SDS-PAGE and stained with Coomassie brilliant blue. **B** Relative abundance of peptidases identified via mass spectrometry. **C** Statistical analysis of the results of mass spectrometry. Wound healing (**D**, **E**, Scale bar: 500 μm) and transwell (**F**, **G**, Scale bar: 100 μm) assays demonstrated that RABL6 overexpression significantly promoted the migratory and invasive capabilities of LSCC cells; however, CHRNA5 knockdown reversed these effects. **H** Western blotting was performed to evaluate RABL6 expression in LSCC cells infected with sh-NC or sh-CHRNA5 and cultured in the presence or absence of nicotine. **I** Differential expression analysis of RABL6 in TCGA cohort. **J** Differential expression analysis of RABL6 in patients with LSCC stratified based on the smoking status in TCGA cohort. **K** Correlation between CHRNA5 and RABL6 expression in TCGA cohort.

Based on the analysis of grayscale values, CHRNA5 was found to modulate the expression of E-cadherin, ZO-1 and N-cadherin, which can affect EMT (Supplementary Fig. 1F). However, the influence of CHRNA5 on EMT appeared to be inconspicuous. These findings suggest that nicotine may promotes LSCC metastasis through other pathways by regulating CHRNA5. To examine the potential mechanisms underlying the stimulation of CHRNA5 by nicotine, IP-MS was performed to identify proteins interacting with CHRNA5. Initially, IP was performed using anti-CHRNA5 antibodies to pull-down-interacting proteins from the lysate of the LSCC cell line AMC-HN8 (Fig. 6A). Subsequently, MS was performed to identify the resulting specific bands and evaluate the relative abundance of proteins (Fig. 6B). As depicted in Fig. 6C, seven proteins were identified as potential interacting partners of CHRNA5. Notably, RABL6 protein levels were significantly higher in the CHRNA5-IP group than in the control group (IgG group), suggesting a potential interaction between CHRNA5 and RABL6.

To examine the functions of RABL6, oe-NC, oe-RABL6 and oe-RABL6 + sh-CHRNA5 lentiviruses were constructed and infected into the LSCC cell lines AMC-HN8 and FD-LSC-1. Western blotting was performed to validate the efficacy of RABL6 overexpression (Supplementary Fig. 2D). Wound healing assay revealed that RABL6 overexpression significantly promoted the migratory capability of LSCC cells (*p* < 0.001; Fig. 6D, E), whereas CHRNA5 knockdown reversed these effects (*p* < 0.0001). Transwell assay demonstrated that RABL6 overexpression notably promoted the migratory (Fig. 6F) and invasive (Fig. 6G) capabilities of LSCC cells, whereas CHRNA5 knockdown reversed these effects. As depicted in Fig. 6H, western blotting revealed that nicotine treatment significantly upregulated RABL6 expression, whereas CHRNA5 knockdown partially inhibited RABL6 expression. The effects of CHRNA5 knockdown were counteracted by those of nicotine. These results suggest that the interaction between CHRNA5 and RABL6 could suppresses the protein degradation of RABL6. In TCGA cohort, RABL6 expression was markedly higher in neoplastic tissues than in their adjacent normal tissues (*p* < 0.01; Fig. 6I). Notably, RABL6 overexpression was predominantly higher among patients with a history of smoking, irrespective of whether they had quit smoking or not (Fig. 6J). In addition, a positive correlation was observed between the expression of RABL6 and CHRNA5 (Fig. 6K).

### CHRNA5 binds to RABL6, primarily to the RABL6-39-279 region, and nicotine modulates LSCC metastasis via the CHRNA5–RABL6 interaction

**Fig. 7:**
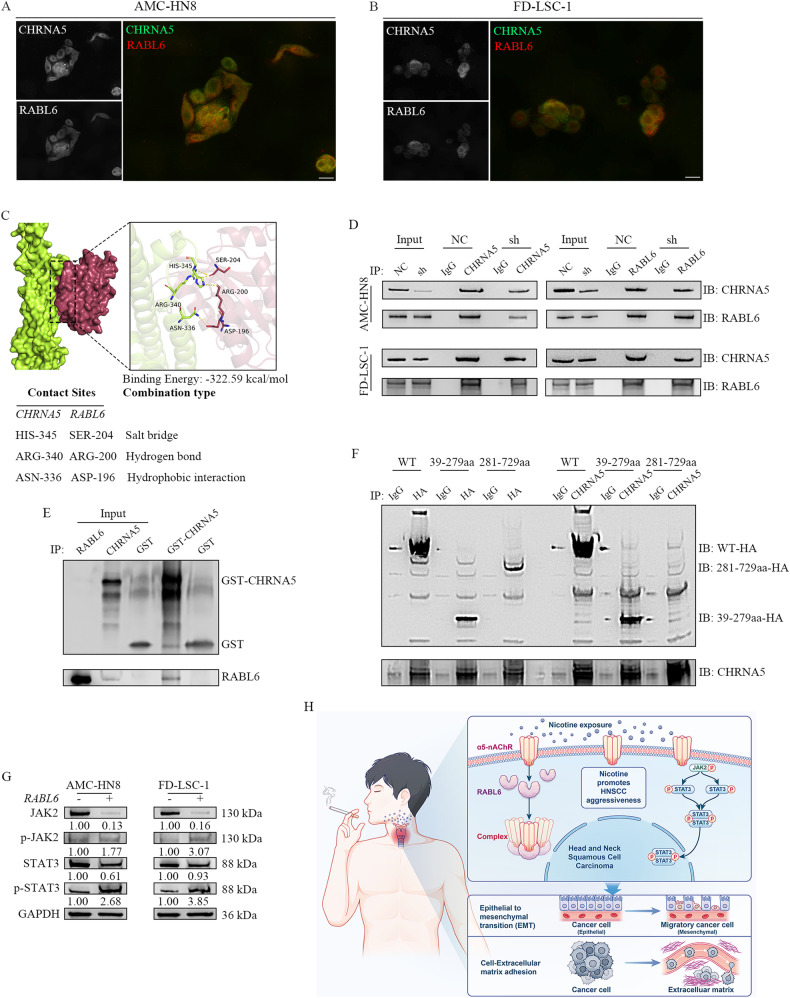
CHRNA5 binds to RABL6, primarily to the RABL6-39-279 region. The subcellular localisation of CHRNA5 and RABL6 proteins was examined in AMC-HN8 (**A**) and FD-LSC-1 (**B**) cells via fluorescence microscopy. Scale bar: 20 μm. **C** Representative images of molecular docking demonstrating the specific residues of the calculated binding site and combination types between CHRNA5 and RABL6. **D** Co-immunoprecipitation of endogenous CHRNA5 and RABL6. Whole-cell extracts from AMC-HN8 and FD-LSC-1 cells and the precipitates were analysed via SDS-PAGE, followed by western blotting with anti-CHRNA5/-RABL6 antibodies. IgG was used as the negative control. **E** GST pull-down assay indicated that recombinant GST-tagged CHRNA5, but not GST, interacted only with RABL6. **F** CHRNA5 specifically interacted with the RABL6-39-279aa region. Co-immunoprecipitation assay demonstrated that endogenous CHRNA5 and RABL6-39-279aa formed a complex in HEK293T cells. **G** Western blotting was performed to evaluate JAK2/STAT3 signalling-related proteins expression in LSCC cells infected with oe-NC and oe-RABL6 lentiviruses. **H** Proposed model of how nicotine exposure activates nicotinic acetylcholine receptors and consequently promotes the metastasis of LSCC.

The mechanisms underlying the interaction between CHRNA5 and RABL6 were further investigated. Immunofluorescence staining revealed that CHRNA5 was abundantly expressed in the cell membrane, whereas RABL6 was mainly localised in the cytoplasm of LSCC cells (Fig. 7A, B). Notably, CHRNA5 and RABL6 were colocalised in LSCC cells, indicating potential interactions between the two proteins in the same cellular environment. Subsequently, molecular docking was performed to predict the binding affinity and binding mode between CHRNA5 and RABL6. As demonstrated in Fig. 7C, the binding energy between CHRNA5 and RABL6 was estimated to be −322.59 kcal/mol. The contact residues between CHRNA5 and RABL6 proteins could form various types of interactions, such as salt bridges, hydrogen bonds and hydrophobic interactions, which effectively enhanced the stability of the CHRNA5–RABL6 complex and the interaction between the two proteins. Co-IP was performed to co-precipitate CHRNA5 and RABL6 proteins in AMC-HN8 (sh-NC and sh-CHRNA5) and FD-LSC-1 (sh-NC and sh-CHRNA5) cells, respectively (Fig. 7D). The results revealed the binding of CHRNA5 to RABL6 in AMC-HN8 and FD-LSC-1 cells and a corresponding decrease in the pulled down RABL6 protein content after CHRNA5 knockdown, which was consistent with the results shown in Fig. 6A. Furthermore, we synthesised and expressed CHRNA5 and RABL6 in vitro and performed GST pull-down assay. As shown in Fig. 7E, both CHRNA5 and RABL6 were detected in the GST-CHRNA5 group, whereas only GST was detected in the control (GST) group, indicating a direct interaction between CHRNA5 and RABL6 proteins.

To delineate the key structural domains responsible for the interaction between CHRNA5 and RABL6 proteins, the UniProt database (http://www.uniprot.org/) was used to predict and construct truncated fragments of the RABL6 protein, including RABL6-39-279aa and RABL6-281-729aa. CHRNA5 was co-transfected with RABL6, RABL6-39-279aa and RABL6-281-729aa into 293 T cells, and the cells were subjected to Co-IP with HA-labelled RABL6 fusion tags. The lysates were precipitated with anti-HA antibodies, and CHRNA5 was pulled down. Similarly, the lysates were precipitated with anti-CHRNA5 antibodies, and RABL6 and RABL6-39-279aa were pulled down, indicating that CHRNA5 mainly binds to the RABL6-39-279aa region (Fig. 7F). As depicted in Supplementary Fig. 2E, the RABL6-39-279aa region had a favourable match with the surface of CHRNA5, which is conducive to the formation of a stable binding effect. The binding energy between CHRNA5 and the RABL6-39-279aa region was estimated to be -309.53 kcal/mol. Moreover, contact residues between CHRNA5 and the RABL6-39-279aa region formed various interactions, such as salt bridges and hydrogen bonds.

Immunofluorescence analysis revealed that nicotine treatment significantly upregulated RABL6 expression in LSCC cells, which was accompanied by a more pronounced co-localisation of RABL6 with CHRNA5 in vitro (Supplementary Fig. 3A). Consistently, compared with mice in the control group, mice in the smoking group had higher protein expression of RABL6 in pulmonary metastatic tissues (Supplementary Fig. 3B) and metastatic lymph nodes (Supplementary Fig. 3C). Notably, the co-localisation of RABL6 with CHRNA5 was more prominent under nicotine stimulation. Furthermore, immunofluorescence staining was performed to detect the expression and localisation of RABL6 in paraffin sections of 44 patients with LSCC (cohort 2). Compared with patients without a smoking history, those with a smoking history (including both former and active smokers) had significantly higher RABL6 expression in tumour tissues and more pronounced co-localisation of RABL6 with CHRNA5 (Supplementary Fig. 3D).

The CHRNA5 promoter harbours a binding site for STAT3, and nicotine induces the proliferation of lung cancer cells by enhancing CHRNA5 expression and activating the JAK2/STAT3 signalling cascade [16]. In this regard, we employed western blotting to verify the involvement of the RABL6/JAK2/STAT3 signalling axis in nicotine-induced LSCC. As demonstrated in Fig. 7G, overexpression of RABL6 significantly activates the JAK2/STAT3 signalling pathway in LSCC cells. Activation of the JAK2/STAT3 pathway facilitates LSCC metastasis by mediating EMT [17–20]. Accordingly, we speculate that nicotine-mediated activation of CHRNA5 promotes the metastasis of LSCC by enhancing its interaction with RABL6 and triggering the activation of the JAK2/STAT3 signalling pathway.

## Discussion

Various chemicals in tobacco can cause changes in cellular genomic and epigenomic landscapes, eventually promoting the proliferative and metastatic capabilities of tumour cells, including lung cancer [21, 22], oesophageal squamous cell carcinoma [23] and breast cancer [24] cells. However, the mechanisms through which smoking affects LSCC cells have not been comprehensively investigated. This study demonstrated that smoking was associated with a high rate of lymph node metastasis in LSCC, which may be the main reason for the poor prognosis of patients with LSCC who smoke. In vivo experiments demonstrated that cigarette smoke enhanced the haematogenous and lymphatic dissemination abilities of LSCC cells. These findings indicate that cigarette smoke is involved in the occurrence, development and metastasis of LSCC. Understanding the impact of smoking on the metastasis of LSCC and its underlying mechanisms is of great significance for the prevention and treatment of nicotine-induced LSCC.

Nicotine, the crucial constituent of cigarette smoke, is closely associated with cancer [6]. It is widely acknowledged that nicotine exerts its biological effects by activating cell surface-expressed nAChRs [25]. In this study, we examined the expression of 15 nAChRs and found that CHRNA7, CHRNB1 and CHRNG were upregulated in some LSCC cells after nicotine stimulation, whereas CHRNA5 was upregulated in all LSCC cell lines. As the most extensively investigated nAChR in cancer research, CHRNA5 has attracted widespread attention owing to its role in cancer metastasis. Activation of CHRNA5 can enhance the migratory and invasive potential of nicotine-stimulated lung cancer [16, 26], hepatocellular carcinoma [27] and melanoma [28] cells. Mechanistically, CHRNA5 mediates the Stat3–Jab1–Csn5 and TGF-β1–Smad signalling pathways to promote the metastasis and EMT of lung cancer cells [16, 26]. CHRNA5 activates the AKT signalling pathway to promote the metastasis and proliferation of prostate cancer cells [29]. In addition, CHRNA5 promotes metastasis and stem cell proliferation in hepatocellular carcinoma by modulating the Hippo signalling pathway [27]. Consistent with previous studies, this study demonstrated that CHRNA5 expression was markedly upregulated in the neoplastic tissues of patients with LSCC who smoked. CHRNA5 overexpression was significantly associated with a poor prognosis in patients with LSCC and was identified as an independent prognostic factor for the disease. In vivo experiments demonstrated that nicotine-induced activation of CHRNA5 promoted the haematogenous and lymphatic metastatic capabilities of LSCC cells and facilitated cell migration and invasion, EMT and cell–ECM adhesion. However, it is noteworthy that the impact of nicotine-induced activation of CHRNA5 on EMT-related proteins was not prominent based on the grayscale values. This finding indicates that nicotine may promote LSCC metastasis via CHRNA5 through other pathways.

As a type of membrane protein, CHRNA5 is involved in the occurrence and development of tumours through various pathways. On the one hand, CHRNA5 can activate multiple signalling pathways, such as TGF-β1–Smad [26], JAK2/STAT3 [16] and Notch1 [28], thus promoting tumour cell proliferation, survival and metastasis. On the other hand, CHRNA5 can affect the sensitivity of tumour cells to radiotherapy and chemotherapy. For example, it can promote sorafenib resistance in liver cancer [27] and reduce the radiosensitivity of oral squamous cell carcinoma [30]. The CHRNA5 promoter had four STAT3 response elements, and the interaction between CHRNA5 and STAT3 facilitates the nicotine-induced proliferation of lung cancer cells [16]. The interaction between CHRNA5 and Ly6E mediates the migration of non-small cell lung cancer cells through the TGF-β1–Smad signalling pathway [26]. In this study, Co-IP and GST pull-down experiments revealed that CHRNA5 directly interacts with RABL6 and that CHRNA5 mainly binds to the RABL6-39-279aa region. This finding reveals a novel signalling pathway and regulatory mechanism underlying the metastasis of LSCC. Drug therapy targeting the abovementioned binding domain may represent a promising option for the treatment of LSCC. This finding provides novel research directions and suggests treatment options for other tumours. Furthermore, we found that nicotine promoted the interaction between RABL6 and CHRNA5 and that nicotine-mediated activation of CHRNA5 regulated the expression of RABL6 protein, promoting the metastatic ability of LSCC cells. The results of IP-MS revealed other potential CHRNA5-interacting proteins, including WRN helicase-interacting protein 1 (WRNIP1), eukaryotic translation elongation factor 1 delta (EEF1D), steroid receptor RNA activator 1 (SRA1), albumin (ALB), zinc finger protein 791 (ZNF791) and phosphodiesterase 4 C (PDE4C). These proteins have not been reported in relation to CHRNA5, and further investigation is warranted to validate their interaction with CHRNA5 and enhance the CHRNA5 protein interaction network.

The RABL6 protein, a member of the Ras family of small GTPases, has attracted substantial interest in the field of oncology owing to its vital role in tumorigenesis. Aberrant expression of RABL6 has been closely associated with the occurrence and progression of various malignancies. RABL6 is markedly upregulated in breast cancer [31, 32], gastric cancer [33], osteosarcoma [34], oesophageal squamous cell carcinoma [35] and pancreatic neuroendocrine tumours [36, 37] and is significantly associated with tumour invasion, metastasis and prognosis. Specifically, RABL6 is highly expressed in breast cancer and promotes cell invasion while stimulating cell proliferation by facilitating entry into the G1 phase and inhibiting apoptosis [31, 32]. RABL6 is upregulated in gastric cancer and is associated with immune regulation and immune cell infiltration. In addition, it promotes tumour cell proliferation, migration, invasion and apoptosis through the circTMC5–miR-361-3p axis [33]. In osteosarcoma, RABL6 acts as an oncogenic driver by regulating the cell cycle by inhibiting Rb protein activity and participating in the p27–Rb1 axis [34, 38]. In oesophageal squamous cell carcinoma, RABL6 promotes cell proliferation and EMT progression by downregulating the epithelial markers E-cadherin and β-catenin, eventually promoting cell invasion and migration [35]. In pancreatic neuroendocrine tumours, RABL6 activates AKT S473 phosphorylation, promoting cell proliferation [36]. In this study, a direct interaction was observed between RABL6 and CHRNA5 in LSCC, and nicotine was found to enhance this interaction. RABL6 overexpression facilitated the migratory and invasive capabilities of LSCC cells, whereas CHRNA5 knockdown attenuated these effects. We speculate that RABL6 interacts with CHRNA5 to form a protein complex, which prevents the degradation of the oncogenic protein RABL6, thereby enabling its sustained expression. Moreover, the CHRNA5–RABL6 protein complex may facilitate the metastasis of LSCC.

The JAK2/STAT3 signalling pathway is closely associated with the occurrence and development of many diseases, particularly cancer. When cells are stimulated by various factors such as growth factors, cytokines or hormones, these signals are transmitted inside the cells, leading to activation of the JAK2/STAT3 signalling pathway [19]. In this study, nicotine was found to mediate the activation of CHRNA5 to enhance its interaction with RABL6 and trigger the activation of the JAK2/STAT3 signalling pathway in LSCC cells. Previous studies have shown that activation of the JAK2/STAT3 signalling pathway can promote the proliferative and metastatic capabilities of LSCC cells [17–20]. Altogether, this study reveals that nicotine upregulates CHRNA5 and activates the RABL6/JAK2/STAT3 signalling axis, eventually promoting the metastasis of LSCC. These findings are consistent with those of a previous study reporting the interaction between CHRNA5 and STAT3 [16].

This study has a few limitations that should be acknowledged. Given the lack of specific inhibitors targeting CHRNA5, we did not address the potential therapeutic implications of targeting the nicotine-mediated CHRNA5–RABL6 interaction in the treatment of LSCC. Future studies should investigate the feasibility and effectiveness of such interventions, which may have significant clinical implications for patients who smoke.

In conclusion, this study reveals that smoking is closely associated with the metastasis and prognosis of LSCC. Inhalation of cigarette smoke can stimulate the haematogenous and lymphatic dissemination of LSCC cells in vivo. Nicotine, the crucial constituent of cigarette smoke, activates the nicotinic acetylcholine receptor CHRNA5, promoting cell migration and invasion, EMT and cell–ECM adhesion in LSCC. CHRNA5 can directly interact with RABL6, predominantly with the RABL6-39-279aa region. Activation of CHRNA5 augments its interaction with RABL6 and stimulates the JAK2/STAT3 signalling pathway to enhance the metastatic ability of LSCC cells (Fig. 7H). Altogether, this study reveals a novel mechanism through which nicotine-mediated CHRNA5–RABL6 interaction promotes the metastasis of LSCC.

## Materials and methods

### Ethics statement

LSCC tissue specimens were obtained from the Department of Otorhinolaryngology at the Eye & ENT Hospital of Fudan University. All participants signed an informed consent form. This study was approved by the clinical research ethics committee of the aforementioned institution (approval number: 2022076) and was conducted in accordance with the guidelines outlined in the Declaration of Helsinki. All animal experiments were performed in compliance with the guidelines and authorisation of the animal centre at the Eye & ENT Hospital of Fudan University.

### Inclusion of patients and specimen collection

This study involved three clinical cohorts: TCGA cohort from The Cancer Genome Atlas (TCGA, https://portal.gdc.cancer.gov) database [39] and cohorts 1 and 2 from the Eye & ENT Hospital of Fudan University. TCGA cohort comprised 527 patients with HNSCC. The baseline clinical information of all patients is provided in Supplementary Table 1. Cohort 1 included 50 pairs of tumour and matched adjacent normal tissues collected from patients with LSCC who underwent surgical treatment at the Eye & ENT Hospital of Fudan University in 2022. The inclusion criteria were as follows: patients with a pathological diagnosis of LSCC, patients who did not undergo preoperative radiotherapy or chemotherapy and patients who signed an informed consent form before surgery. Tumour tissue samples were collected from the centre of the tumour at a low temperature, whereas the matched adjacent normal tissues were collected from the mucosa 1.5 cm outside the tumour edge. The tissues were aliquoted, labelled and stored at −80 °C until further use. The baseline clinical information of patients is presented in Supplementary Table 1. Cohort 2 consisted of 44 paraffin-embedded LSCC tissues collected from patients who underwent surgical treatment at the Eye & ENT Hospital of Fudan University in 2009–2010. The tissue sections had a thickness of 4-6 μm, and the basic clinical information of patients is presented in Supplementary Table 1.

### Cell culture

The LSCC cell lines AMC-HN8, FD-LSC-1, FD-LS-6 and Tu686 were utilised in this study. AMC-HN8 cell lines were generously given by Professor Kim SY of Samsung Medical Center, Korea, while Tu686 cell lines was obtained from Cell Bank of the Shanghai Institute of Cells, Chinese Academy of Science (Shanghai, China). AMC-HN8 and Tu686 cell lines were maintained in RPMI-1640 (HyClone, USA) supplemented with 10% fetal bovine serum (FBS) and 1% penicillin–streptomycin, and incubated in a humidified atmosphere with 5% CO_2_ at 37 °C. FD-LSC-1 and FD-LS-6 cell lines were obtained from our lab [40, 41] and were cultured in BEGM (CC-3170, Lonza, USA) supplemented with 1% penicillin-streptomycin, and maintained at 37 °C with 5% CO_2_. The culture medium was changed every 2-3 days and cells were passaged when they reached 80% confluency.

### RNA isolation and quantitative real-time PCR

The complete RNA was isolated using the TRIzol reagent (Thermo Fisher) and reverse transcription was then carried out according to the manufacturer’s protocol. The quantitative real-time PCR assay was performed on the ABI 7500 Real-Time PCR system (Thermo Fisher), and the housekeeping gene GAPDH was used as an internal control. The primers were designed using the NCBI Primer-BLAST and synthesised by Sangon Biotech (Shanghai, China). The sequences of all primers are listed in Supplementary Table 2.

### Western blot, immunofluorescence staining and immunohistochemical staining

The protein expression levels were determined using western blot analysis. Briefly, cell lysates were prepared using RIPA buffer and quantified using a BCA protein assay kit. Equal amounts of protein were loaded onto SDS-PAGE gels and transferred onto PVDF membranes. The membranes were then incubated with primary antibodies overnight at 4 °C, followed by incubation with secondary antibodies. The protein bands were visualised using enhanced chemiluminescence and quantified using densitometry analysis. GAPDH was used as a loading control to normalise the protein expression levels. Cell fractions (nucleus, cytoplasm and membrane) were extracted using a Nuclear and Cytoplasmic Protein Extraction Kit (abs9346-50T, Absin Bioscience Inc., Shanghai, China).

The immunofluorescence staining experiment was performed following standard protocols. The samples were fixed with paraformaldehyde, permeabilized with Triton X-100 and blocked with 5% BSA. The primary antibodies were incubated overnight at 4 °C, followed by incubation with the secondary antibodies conjugated with fluorescent dyes. The nuclei were counterstained with DAPI, and images were captured using a fluorescence microscope. The fluorescence intensity was quantified using ImageJ software.

Immunohistochemical staining was performed to assess the expression of the target proteins in tissue specimens. In brief, the tissue sections were subjected to deparaffinization, rehydration, and heat-induced epitope retrieval. Following blocking of the endogenous peroxidase activity, primary antibodies targeting the proteins of interest were incubated overnight at a temperature of 4 °C. Slides were then incubated with a secondary antibody and developed with DAB. Details of all antibodies are listed in Supplementary Table 3.

### Proliferation, migration, and invasion assays

Cells were seeded in 96-well plates at a density of 2000 cells per well for a duration of 1–5 days. Cell viability was measured utilising the CCK-8 assay. 10 μL CCK-8 solution was added to each well and incubated for 1 h. The optical density at 450 nm was gauged utilising a microplate reader.

For cell migration ability, we used the scratch assay by creating a straight scratch in a cell monolayer using a 100-μL sterile pipette tip. The movement of cells into the wound area was monitored under a microscope at 0 and 48 h.

To test cell migration and invasion, we used transwell assays. For the migration assay, we seeded cells with serum-free medium in the upper chamber. The lower chamber contained medium supplemented with 10% FBS as a chemoattractant. After 72 h of incubation, the transwell inserts were washed with PBS, fixed with 4% paraformaldehyde and stained with crystal violet to visualise the migrated or invaded cells. For the invasion assay, we seeded cells in transwell inserts pre-coated with Matrigel (BD Biosciences) and observed the cells that had invaded through the Matrigel and the membrane under a microscope.

### Cell adhesion assay

Cell-to-extracellular matrix (ECM) adhesion was assessed using the CytoSelect™ 48-well Cell Adhesion Assay kit (CBA-070, Cell Biolabs) according to the manufacturer’s instructions. Briefly, cells were seeded in a 48-well plate at a density of 1.5 × 10^5^ cells/well and incubated for 60 min. After the unbound cells were removed, the adherent cells were stained with Cell Staining Solution for 10 min at room temperature. Any residual stain was removed by washing the plate with deionised water, and the plate was allowed to air dry. Thereafter, the plate was incubated with an extraction solution on an orbital shaker for 10 min, and optical density (OD) was measured at 560 nm. Bovine serum albumin (BSA) was used as a negative control.

### Immunoprecipitation–mass spectrometry

Immunoprecipitation–mass spectrometry (IP-MS) was performed to identify proteins that interacted with the antibody of interest in cells. The cell lysates were incubated with the primary antibody and protein A/G beads overnight. Eluted proteins were digested and analysed via mass spectrometry.

### Co-immunoprecipitation

Co-immunoprecipitation (Co-IP) was performed to examine protein–protein interactions in cell samples. Cells were lysed in a solution containing protease inhibitors. The lysate was incubated with the primary antibody of interest and protein A/G beads overnight at 4 °C with gentle agitation. The following day, the antibody–bead–protein complex was washed with lysis buffer and eluted using an elution buffer. The eluted proteins were subjected to western blotting assays.

### Glutathione-S-transferase (GST) pull-down

Prokaryotic expression plasmids were constructed including GST-CHRNA5, His-RABL6, and transformed into *Escherichia coli* competent cell BL21. GST-CHRNA5 protein was incubated with Glutathione high-capacity magnetic agarose beads (G0924, Sigma) and His-RABL6 protein at 4 °C overnight. Then protein complexes were eluted by Elution Buffer and then subjected to SDS-PAGE and analysed by western blot assays.

### Molecular docking

Protein configuration was acquired from the UniProt database (http://www.uniprot.org/) [42] and pre-processed using the MOE (version 2019.1) software. Molecular docking was performed using the HDOCK server, and the conformation with the most pessimistic score for optimisation was designated using the MOE (version 2019.1) software. The results were visualised using the Pymol (version 2.1) software.

### Establishment of mouse models of pulmonary colonisation

Mouse models of pulmonary colonisation were established for the assessment of metastasis in vivo. Briefly, six-week-old male nude mice (Chengqin Bio-Technology Co., Ltd, Shanghai, China) were injected with 1.5 × 10^6^ luciferase-labelled AMC-HN8 cells in 100-μL PBS via the tail vein and were randomly divided into different groups [43]. Mice in the smoking group were housed in a chamber with smoke exposure for 45 min twice per day for 5 days per week for 8 weeks [44], whereas mice in the control group were exposed to ambient air. All mice were monitored daily for general health, and tumour growth, distribution and metastasis were evaluated via bioluminescence imaging (BLI) using an in vivo imaging system. After 8 weeks of injection, the mice were euthanised and their lungs were excised. The lung tissues were fixed in 10% neutral buffered formalin, embedded in paraffin and sectioned (4-μm thickness) for histopathological assessment.

### Establishment of mouse models of lymphatic metastasis

Mouse models of lymphatic metastasis were established by injecting the right hind foot pads of mice with 1.5 × 10^6^ luciferase-labelled AMC-HN8 cells in 50-µL PBS [45]. Mice were randomly divided into different groups, and the progression of tumour growth and lymph node metastasis was monitored via BLI. The mice were euthanised 8 weeks after injection, and lymph nodes were collected for histological analysis.

### Statistical analysis

The GraphPad Prism 7 software was used for statistical analysis and for plotting graphs. The Kaplan–Meier (KM) plotter was used to plot survival curves, which were compared using the log-rank test. Pearson chi-squared test was performed to analyse the correlation between clinical pathological parameters. Student’s *t*-test was employed to evaluate intergroup differences. Univariate and multivariate Cox regression analyses were performed to identify independent prognostic factors for LSCC. Spearman correlation analysis was employed to assess the correlation between variables. The experiments were repeated three times to ensure the reliability of the results. A *p*-value of < 0.05 indicated statistically significant differences.

### Supplementary information

supplementary material legends

Original Data File

Supplementary Figure 1

Supplementary Figure 2

Supplementary Figure 3

Supplementary Table 1

Supplementary Table 2

Supplementary Table 3

## Data Availability

All the source data supporting the findings of this study are available from the corresponding author upon reasonable request.
