# Supplementary material for: Nicotine-induced activation of cholinergic receptor nicotinic alpha 5 subunit mediates the malignant behaviours of laryngeal squamous epithelial cells by interacting with RABL6
Source: Cell Death Discov. 2024 Jun 15;10:286. doi: 10.1038/s41420-024-02051-x (PMC11180178; doi:10.1038/s41420-024-02051-x)
Supplement: Supplementary file 1 — supplementary material legends [file 41420_2024_2051_MOESM1_ESM.docx]

***Supplementary Figure 1:*** *(A) Construction of luciferase-labelled AMC-HN8 cells. Scale bar: 500 μm. (B) Representative images of IHC staining of inguinal lymph nodes with pan-cytokeratin. Scale bar: 20 μm. (C) Western blotting was performed to evaluate EMT-related proteins (E-cadherin, N-cadherin, Slug, ZO-1, Claudin-1 and β-catenin) expression after nicotine stimulation in LSCC cells. (D) Protein expression of CHRNA5 in the subcellular region after nicotine stimulation. GAPDH served as the cytoplasmic and cytomembrane internal reference, whereas histone was used as the nuclear internal reference. (E) Construction of GFP-labelled LSCC cells infected with sh-CHRNA5 and sh-NC. Scale bar: 100 μm. (F) Western blotting was performed on EMT-related proteins (E-cadherin, N-cadherin, and ZO-1) in LSCC cells with or without transduced sh-CHRNA5 lentiviral vector and subsequent coculture with nicotine. (G) Representative images of the gross appearance of lung dissected in nude mice.*

***Supplementary Figure 2:*** *(A) Differential expression analysis of CHRNA5 in cohort 1. (B) Differential expression analysis of CHRNA5 in patients with LSCC stratified based on the smoking status in cohort 1. (C) Correlation between CHRNA5 and the number of pack-years in cohort 1. (D) Western blotting was performed to examine the protein expression of RABL6, respectively, in AMC-HN8 and FD-LSC-1 cells infected with oe-NC and oe-RABL6 lentiviruses. (E) Representative images of molecular docking demonstrating the specific residues of the calculated binding site and combination types between CHRNA5 and RABL6-39-279aa region.*

***Supplementary Figure 3:*** *(A) Immunofluorescence staining revealed that nicotine treatment significantly upregulated RABL6 (red) expression in LSCC cells, which was accompanied by a more pronounced co-localisation of RABL6 with CHRNA5 (green) in LSCC cells. Scale bar: 20 μm. Immunofluorescence staining revealed that mice in the smoking group had higher protein expression of RABL6 (red) in pulmonary metastatic tissues (B) and metastatic lymph nodes (C), and the co-localisation with CHRNA5 (green) is more pronounced. Scale bar: 20 μm. (D) Smoking patients with LSCC had significantly higher RABL6 expression in tumour tissues and more pronounced co-localisation of RABL6 (red) with CHRNA5 (green). Scale bar: 20 μm.*

***Supplementary Table 1: Clinicopathological features of clinical cohorts***

***Supplementary Table 2: The sequences of all primers used in this study.***

***Supplementary Table 3: The antibodies used in this study.***
