# Supplementary material for: Nicotine-induced activation of cholinergic receptor nicotinic alpha 5 subunit mediates the malignant behaviours of laryngeal squamous epithelial cells by interacting with RABL6
Source: Cell Death Discov. 2024 Jun 15;10:286. doi: 10.1038/s41420-024-02051-x (PMC11180178; doi:10.1038/s41420-024-02051-x)

Figure 2J

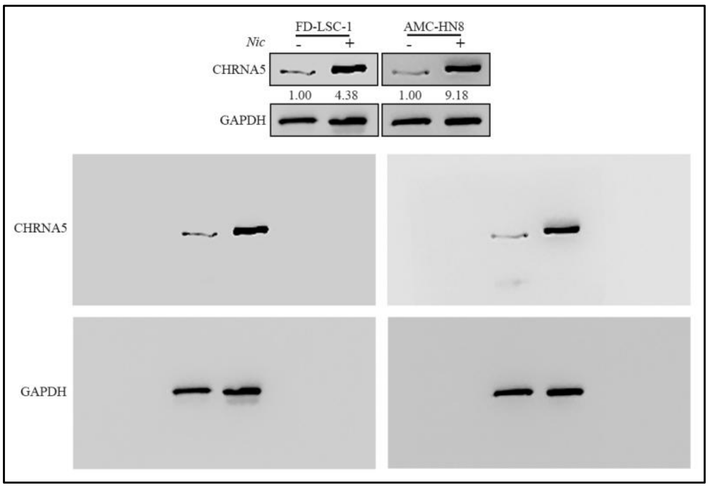

Figure 3B

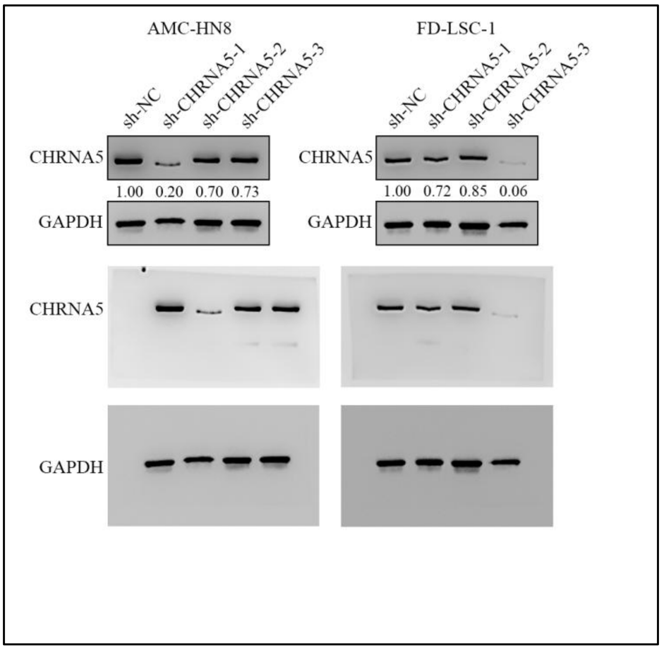

Figure 6H

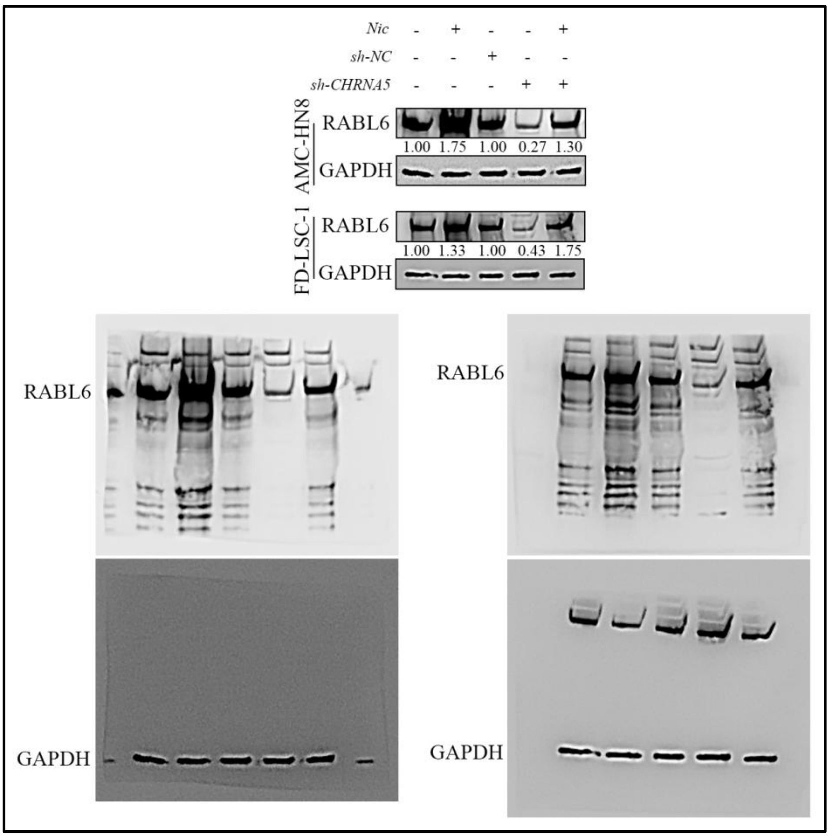
Nic - + - - +
sh-NC - - + - -
sh-CHRNA5 - - - + +
AMC-HN8
RABL6
1.00 1.75 1.00 0.27 1.30
GAPDH
FD-LSC-1
RABL6
1.00 1.33 1.00 0.43 1.75
GAPDH
RABL6
RABL6
GAPDH
GAPDH

Figure 7D

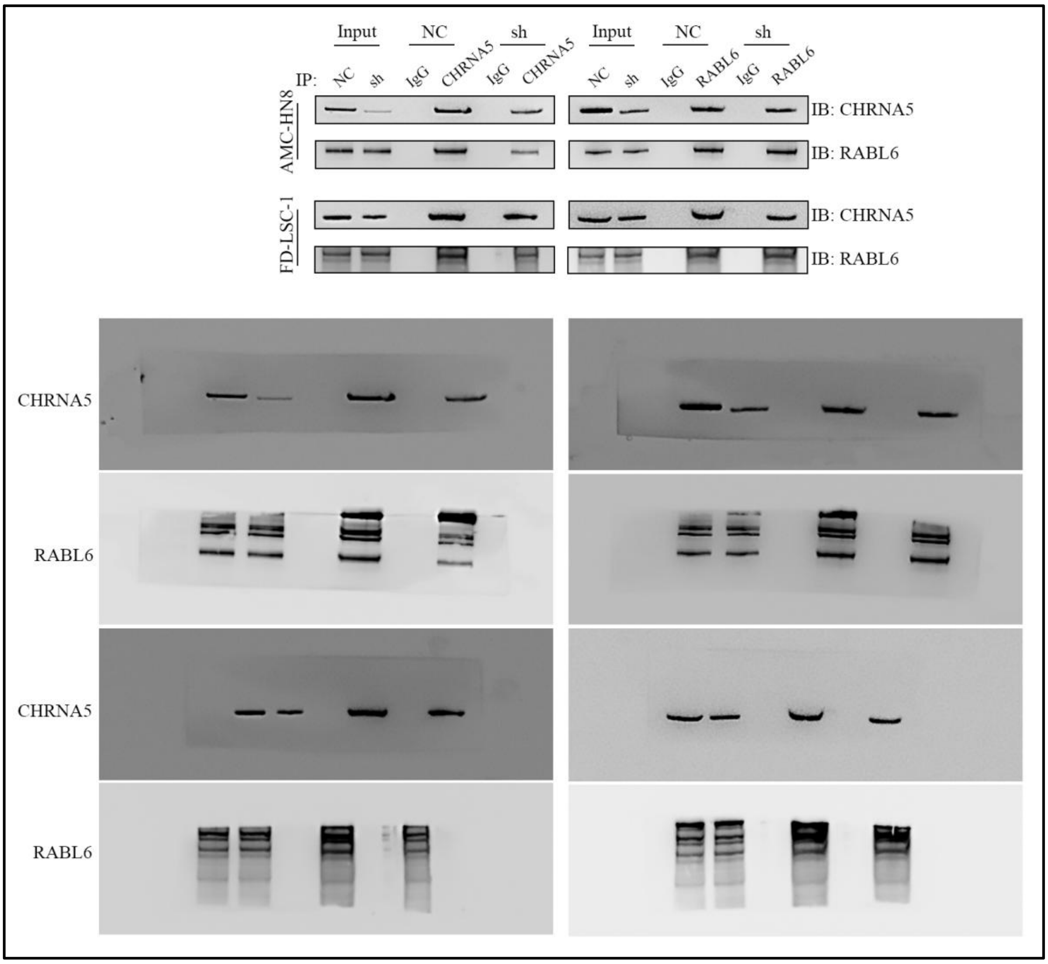
Input
NC
sh
IP: NC sh IgG CHRNA5 IgG CHRNA5
NC sh IgG RABL6 IgG RABL6
AMC-HN8
FD-LSC-1
IB: CHRNA5
IB: RABL6
IB: CHRNA5
IB: RABL6
CHRNA5
RABL6
CHRNA5
RABL6

Figure 7E

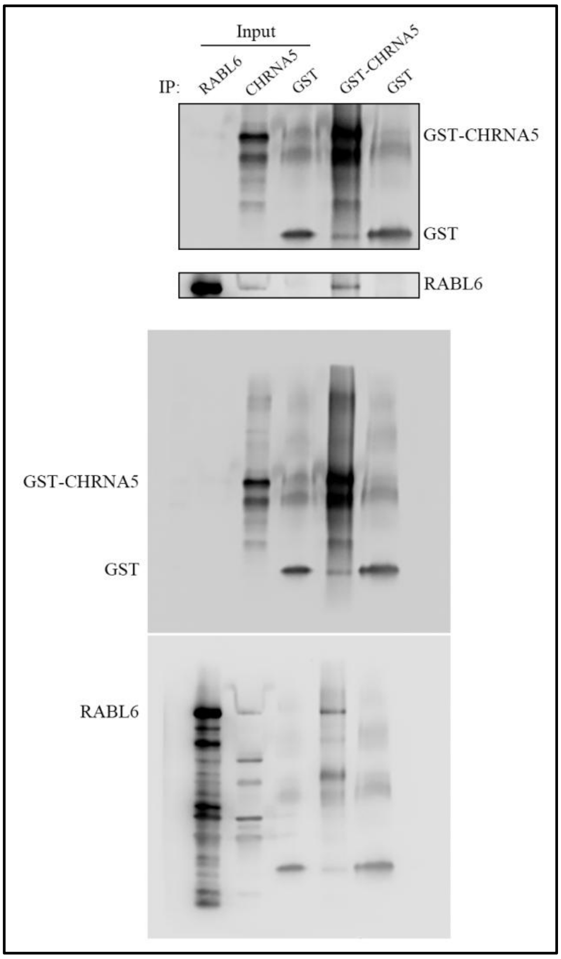
Input
IP:
RABL6
CHRNA5
GST
GST-CHRNA5
GST
GST-CHRNA5
GST
RABL6
GST-CHRNA5
GST
RABL6

Figure 7F

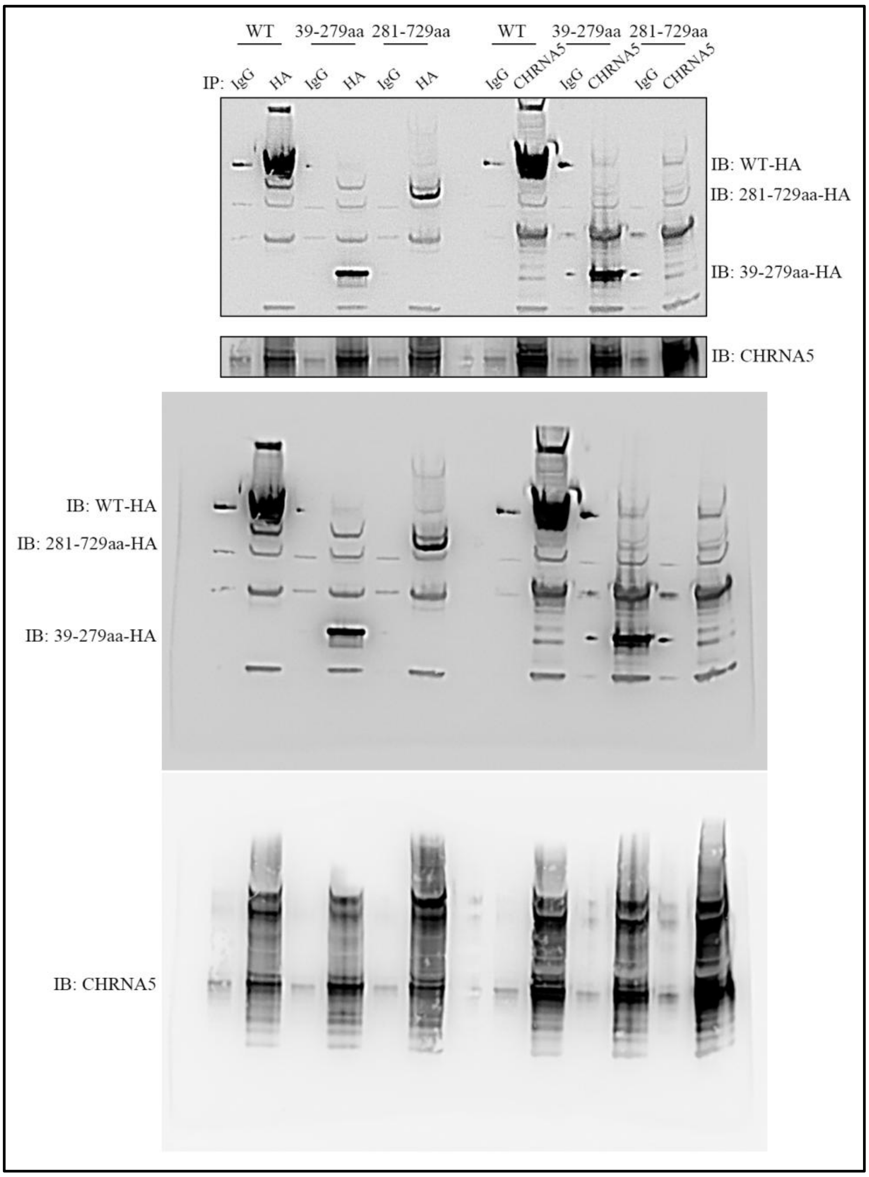

WT
39-279aa
281-729aa
WT
39-279aa
281-729aa
IP: IgG HA IgG HA IgG HA IgG CHRNA5 IgG CHRNA5 IgG CHRNA5
IB: WT-HA
IB: 281-729aa-HA
IB: 39-279aa-HA
IB: CHRNA5
IB: WT-HA
IB: 281-729aa-HA
IB: 39-279aa-HA
IB: CHRNA5

Figure 7G

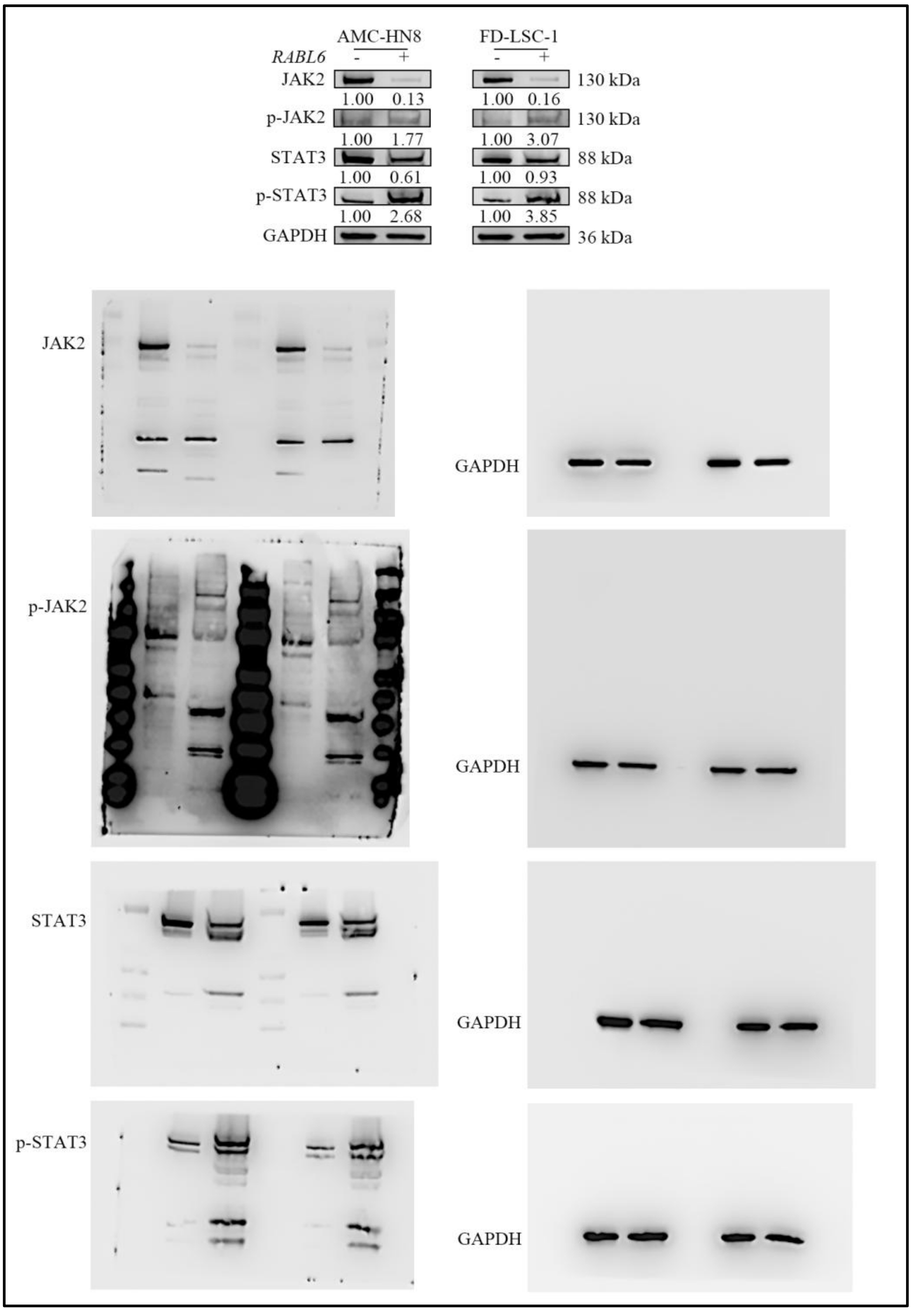
AMC-HN8
FD-LSC-1
RABL6
-
+
JAK2
1.00 0.13
1.00 0.16
130 kDa
p-JAK2
1.00 1.77
1.00 3.07
130 kDa
STAT3
1.00 0.61
1.00 0.93
88 kDa
p-STAT3
1.00 2.68
1.00 3.85
88 kDa
GAPDH
36 kDa
JAK2
GAPDH
p-JAK2
GAPDH
STAT3
GAPDH
p-STAT3
GAPDH

Supplementary Figure 1C

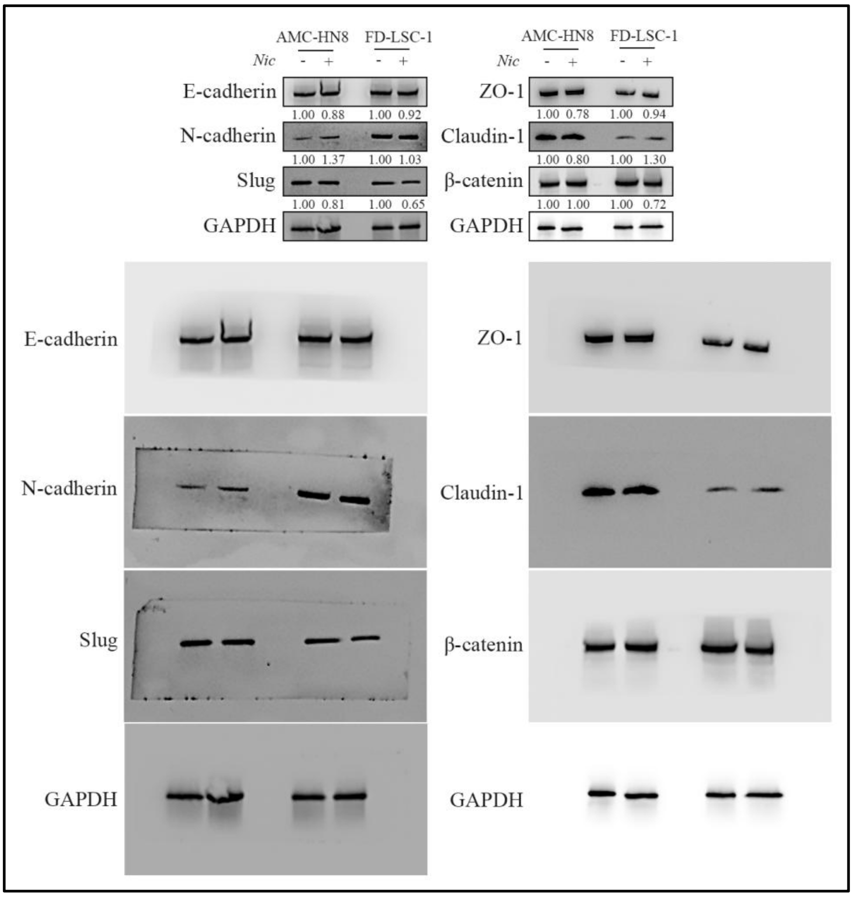

Supplementary Figure 1D

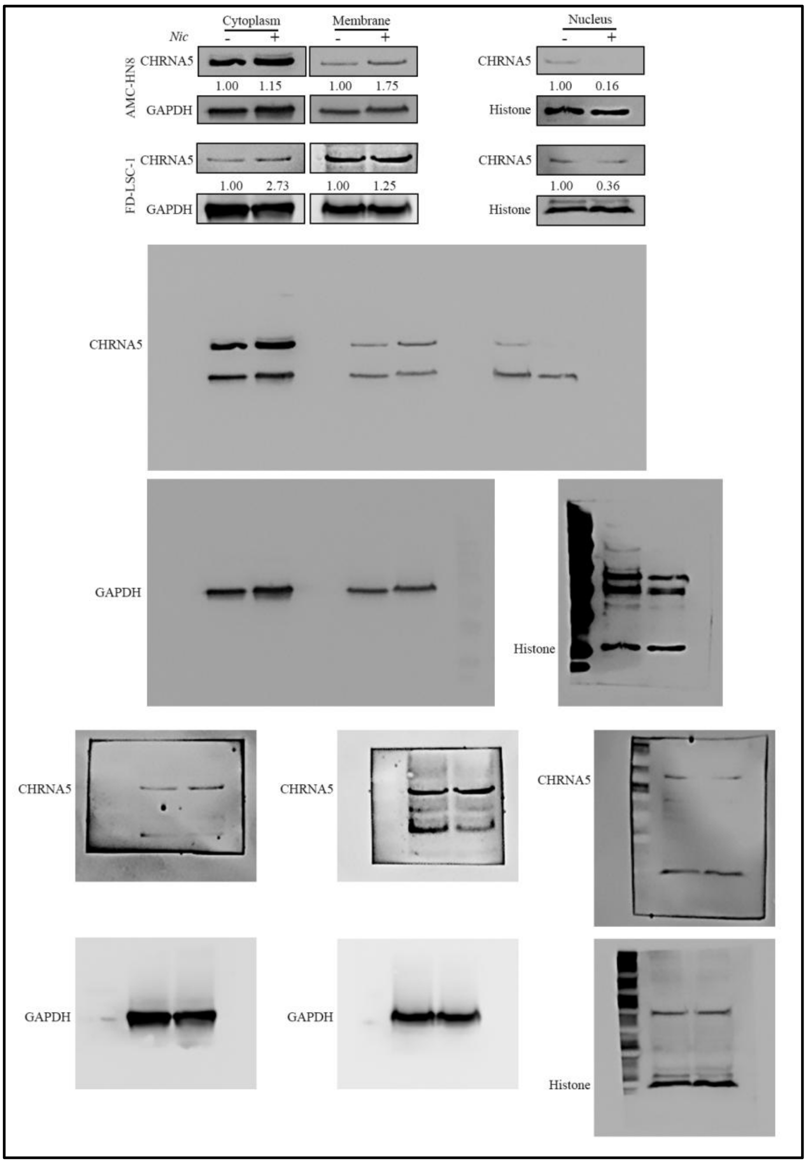

Supplementary Figure 1F

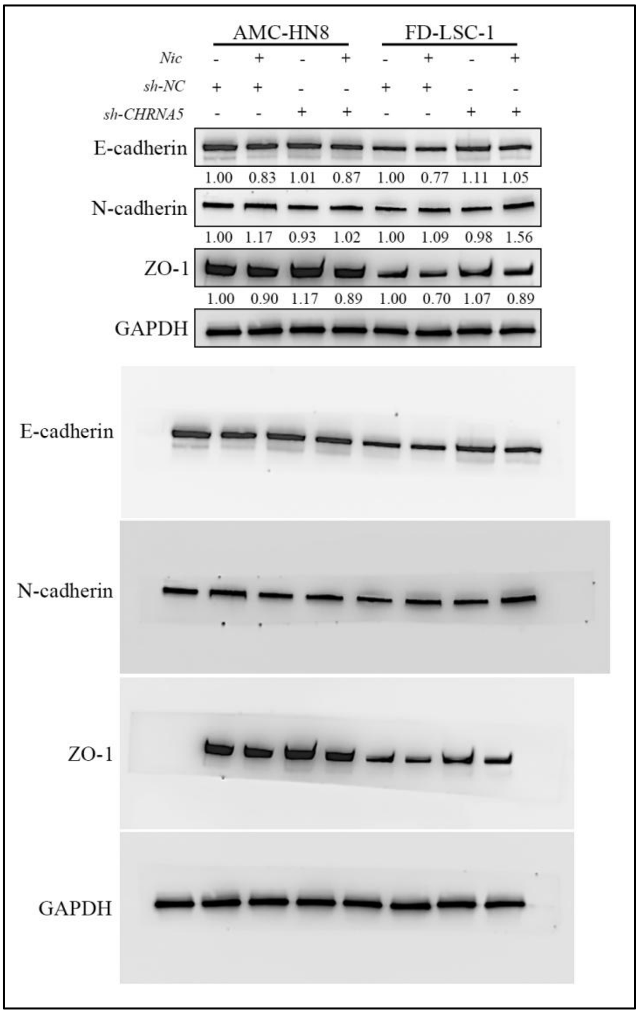

Supplementary Figure 2D

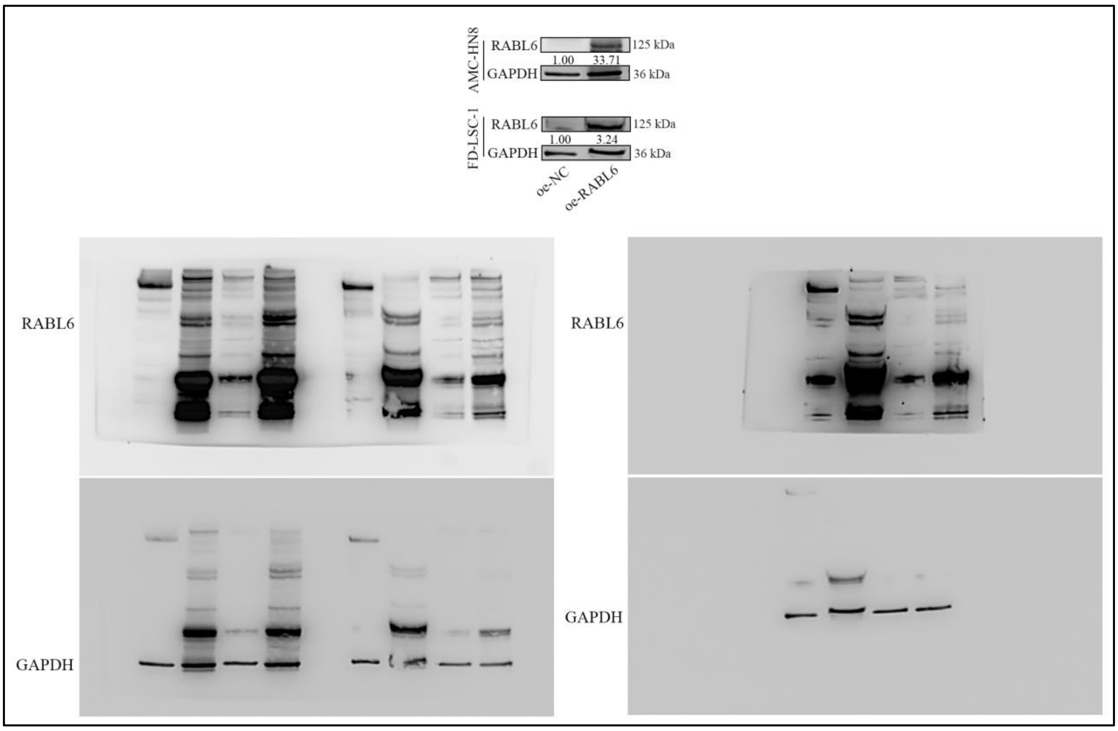

Supplement: Supplementary file 2 — Original Data File [file 41420_2024_2051_MOESM2_ESM.pdf]
