# Supplementary figures and images for: Nicotine-induced activation of cholinergic receptor nicotinic alpha 5 subunit mediates the malignant behaviours of laryngeal squamous epithelial cells by interacting with RABL6
Source: Cell Death Discov. 2024 Jun 15;10:286. doi: 10.1038/s41420-024-02051-x (PMC11180178; doi:10.1038/s41420-024-02051-x)

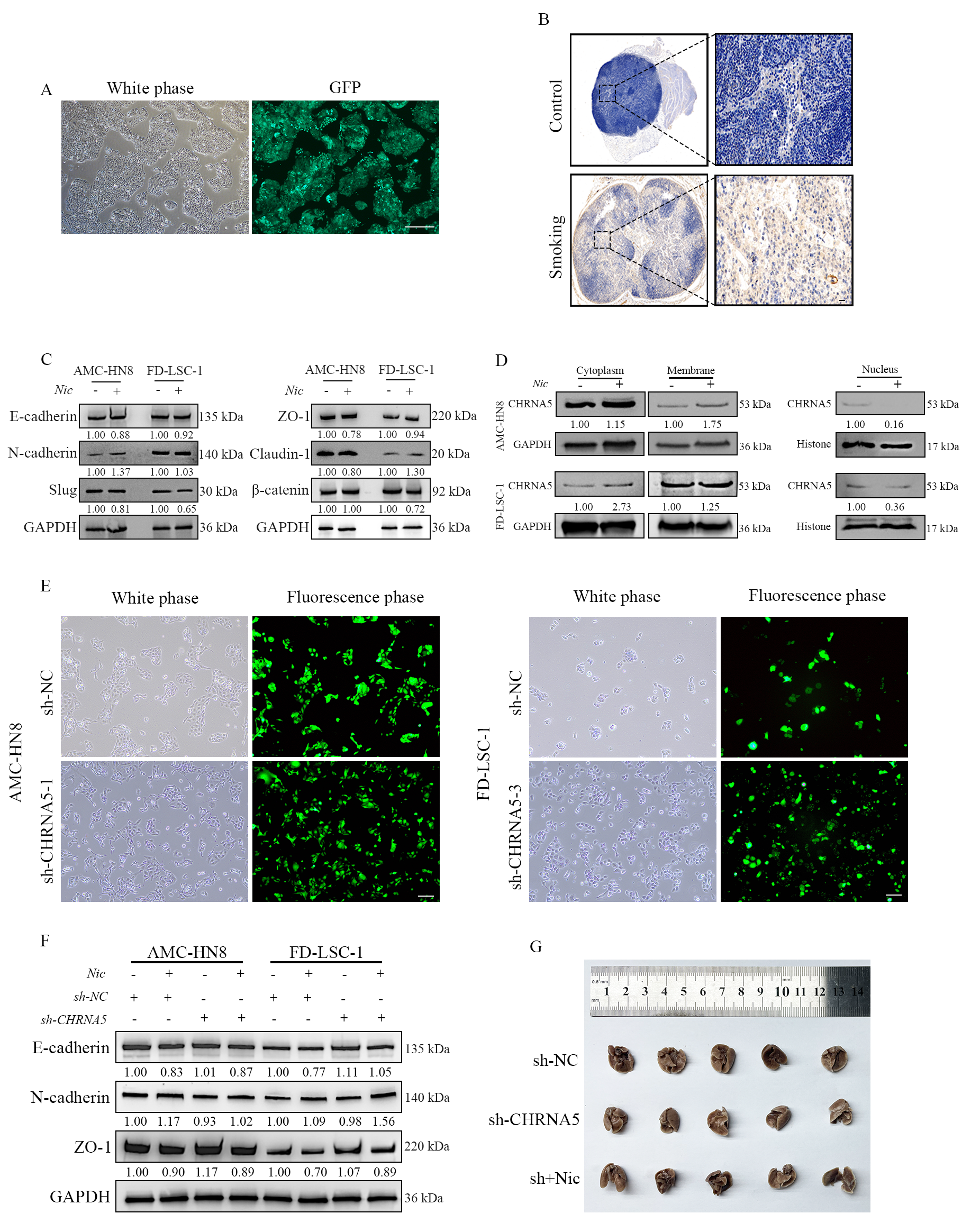

Supplement: Supplementary file 3 — Supplementary Figure 1 [file 41420_2024_2051_MOESM3_ESM.tif]

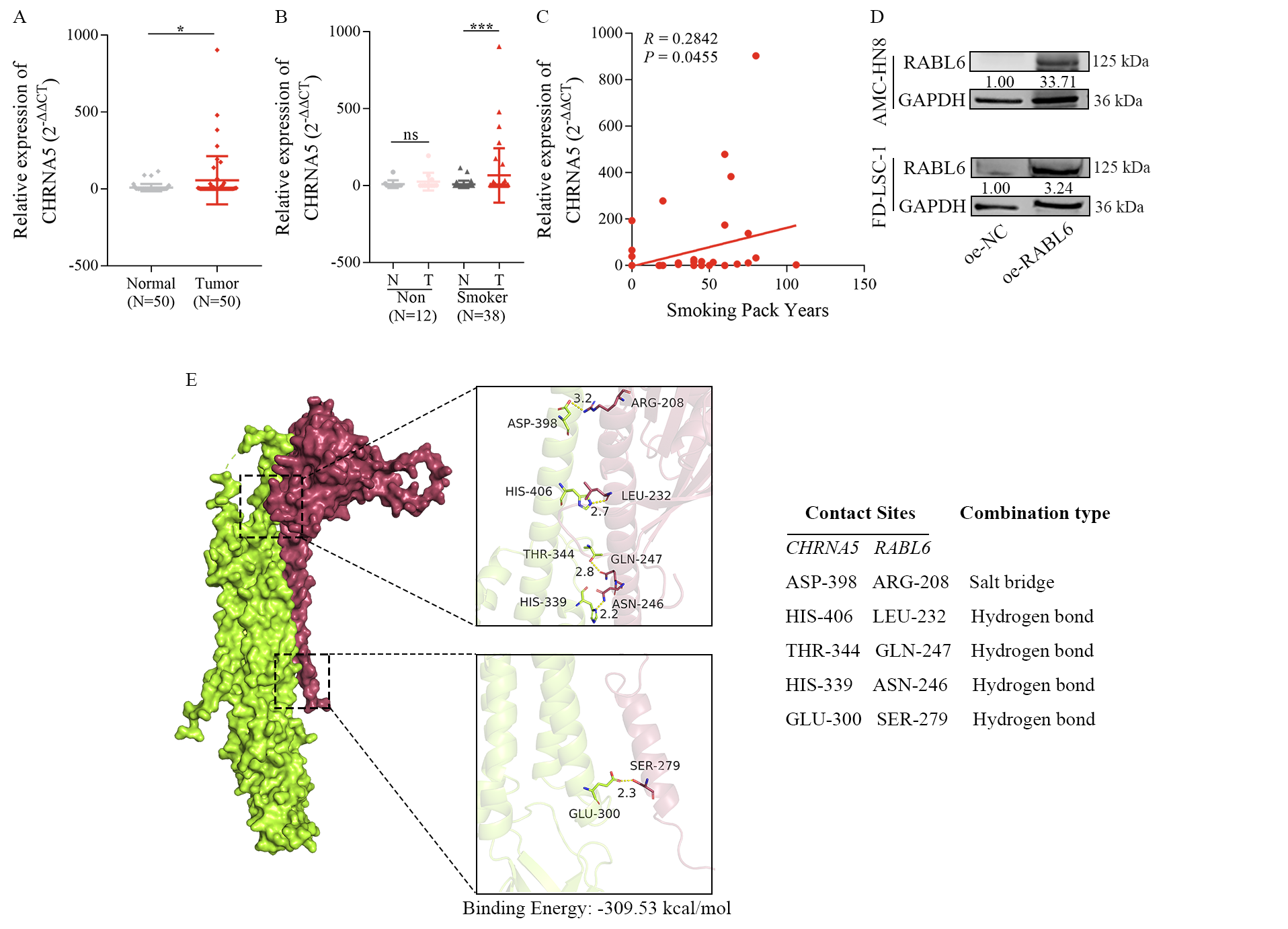

Supplement: Supplementary file 4 — Supplementary Figure 2 [file 41420_2024_2051_MOESM4_ESM.tif]

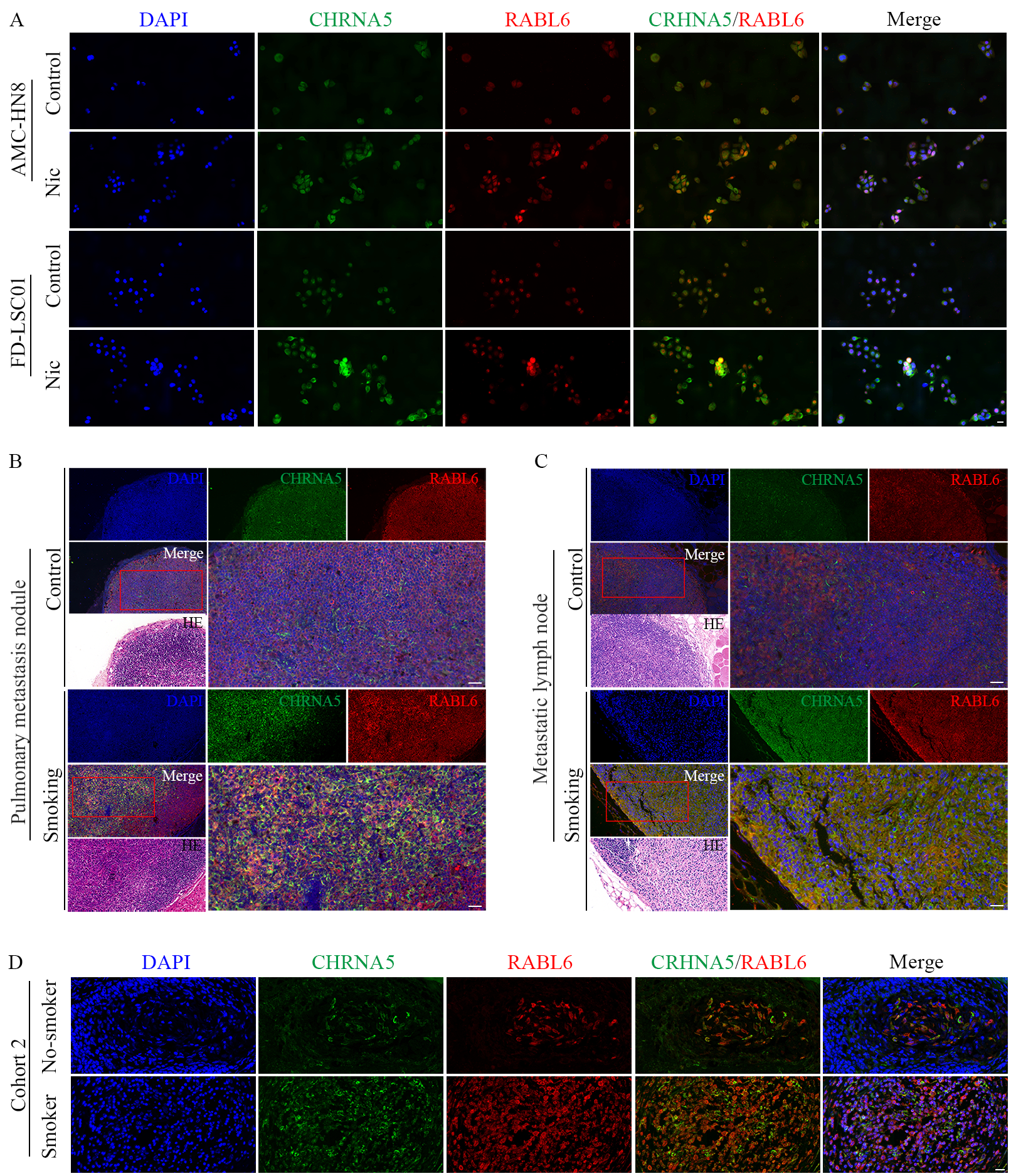

Supplement: Supplementary file 5 — Supplementary Figure 3 [file 41420_2024_2051_MOESM5_ESM.tif]
